# The Effects of Terlipressin on Regional Hemodynamics and Kidney Function in Experimental Hyperdynamic Sepsis

**DOI:** 10.1371/journal.pone.0029693

**Published:** 2012-02-15

**Authors:** Ken Ishikawa, Li Wan, Paolo Calzavacca, Rinaldo Bellomo, Michael Bailey, Clive N. May

**Affiliations:** 1 Howard Florey Institute, University of Melbourne, Parkville, Victoria, Australia; 2 Department of Intensive Care and Department of Medicine, Austin Health, Melbourne, Victoria, Australia; 3 School of Epidemiology and Preventive Medicine, Australian and New Zealand Intensive Care Research Centre, Monash University, Melbourne, Victoria, Australia; University of Sao Paulo Medical School, Brazil

## Abstract

**Background and Aims:**

Although terlipressin (TP) may improve renal function in cirrhotic patients, its use in sepsis remains controversial due to concerns about regional ischemia. We investigated the effects of TP on regional hemodynamics and kidney function in experimental hyperdynamic sepsis.

**Methods:**

We studied thirteen merino ewes in a university physiology laboratory using a randomized controlled cross over design. We implanted flow probes around the pulmonary, circumflex coronary, superior mesenteric, renal and iliac arteries. We injected live *Escherichia coli* and induced hyperdynamic sepsis. We treated animals with either bolus vehicle or a single dose of TP (sTP = 1 mg). In a second group, after 1 mg of TP, two additional bolus injections (mTP) of 0.5 mg were given at 2 hourly intervals.

**Main Results:**

sTP (1 mg) significantly increased mean arterial pressure (MAP) (74 to 89 mmHg; P<0.0001) creatinine clearance (31 to 85 mL/min; P<0.0001) and urine output (24 to 307 mL/hr) (P<0.0001). However, it decreased CO (5.7 to 3.9 L/min; p<0.0001), coronary blood flow (CBF) (43 to 32 mL/min; p<0.0001) and mesenteric blood flow (MBF) (944 to 625 mL/min; p = 0.004) and increased blood lactate (2.1 to 4.0 mmol/L; p<0.0001). Extra doses of TP caused little additional effect.

**Conclusions:**

In hyperdynamic sepsis, bolus TP transiently improves MAP and renal function, but reduces CO, CBF and MBF, and increases blood lactate. Caution should be applied when prescribing bolus TP in septic patients at risk of coronary or mesenteric ischemia.

## Introduction

Severe sepsis is a major cause of death in critically ill patients and its incidence is increasing [1]. In septic patients, catecholamines are frequently used to improve perfusion pressure [2], [3]. However, vascular hyporeactivity to catecholamines is common [4], [5] suggesting the need to either use other vasopressor agents or combine catecholamines with other vasopressor agents. For example, arginine vasopressin (AVP), which acts on vascular smooth muscle via V1 receptors [6], has been used as an additional vasoactive drug in patients with septic shock [2], [7]. Such addition is postulated to also improve kidney dysfunction in patients with septic shock [8]. However, an agent with longer half life and greater V1 selectivity would appear desirable.

Terlipressin (TP) is a synthetic analogue of AVP, with greater selectivity for the V1 receptor and a longer half-life. TP has proved efficacious in the treatment of the hepatorenal syndrome [9], another condition characterized by marked systemic vasodilatation and renal vasoconstriction (a state similar to sepsis). Given such biological properties and efficacy in the hepatorenal syndrome, intermittent intravenous boluses of TP have been used to restore blood pressure in septic shock patients [4], [10]. However, serious adverse events have been reported and there is uncertainty over its ability to replicate the potential beneficial effects of AVP [11]–[13] and its safety in this setting. Such uncertainty derives, in part, from the lack of controlled experimental data on its regional effects.

We hypothesized that TP would reduce vital organ blood flow in mammalian hyperdynamic sepsis and thus examined its effects (both single and repeated administration) on systemic and regional hemodynamics and kidney function in a sheep model of hyperdynamic sepsis.

## Methods

### Animal preparation

Experiments were completed on 13 adult Merino ewes (female sheep) (28–37 kg) housed in individual metabolic cages, with free access to food and water. The experimental procedures were approved by the Animal Experimental Ethics Committee of the Howard Florey Institute under guidelines laid down by the National Health and Medical Research Council of Australia.

The animals underwent three sterile surgical procedures under anesthesia at intervals of 2 weeks. Anesthesia was induced with intravenous sodium thiopentone (15 mg/kg) for intubation with an endotracheal tube (cuffed size 10). Maintenance of anesthesia was by means of oxygen/air/isoflurane (1.5–2.0%). Fractional inspired oxygen was altered to maintain PaO_2_ at approximately 100 mmHg, and ventilation was controlled to maintain PaCO_2_ at approximately 40 mmHg. First, double carotid arterial loops were created to facilitate subsequent arterial cannulation. Second, transit time flow probes (Transonics Systems, Ithaca, NY, USA) were placed on the pulmonary artery and the left circumflex coronary artery. Finally, transit time flow probes were placed on the left renal artery, the superior mesenteric artery and the left external iliac artery.

The animals were allowed at least two weeks to recover before the start of experiments. In all operations, animals were treated with intramuscular antibiotics (900 mg, Ilium Propen; procaine penicillin, Troy Laboratories Ptd Ltd, Smithfield, NSW, Australia or Mavlab, Qld, Australia) at the start of surgery and then for 2 days postoperatively. Post-surgical analgesia was maintained with intramuscular injection of flunixin meglumine (1 mg/kg) (Troy Laboratories or Mavlab, Qld, Australia) at the start of surgery, then 4 and 16 hours post-surgery.

The day before the experiment, a Tygon catheter (Cole-Parmers; Boronia, Australia; ID 1.0 mm, OD 1.5 mm) was inserted into the carotid arterial loop to measure arterial pressure. A polythene catheter (Portex®; Smiths Medical International Ltd. Hythe, Kens UK; ID 1.19 mm, OD 1.70) was inserted into a jugular vein for measurement of central venous pressure and infusion. Analog signals (mean arterial pressure (MAP), heart rate, cardiac output (CO) and vital organ blood flow (coronary blood flow: CBF, mesenteric blood flow: MBF, iliac blood flow: IBF, and renal blood flow: RBF) were collected on computer using a customized data-acquisition system (Labview; National Instruments; Austin, Texas, USA). The data were recorded at 100 Hz for 10 second every minute during experiments. Total peripheral conductance (TPC = CO/MAP) regional conductance (organ conductance = organ blood flow/MAP) and stroke volume (SV = CO/HR) were calculated.

Blood samples were taken the day before the induction of sepsis, at the end of the sepsis control period, and every two hours during intervention period for six hours. Urine was collected and sampled every two hours throughout the experiment from the bladder catheter using an automated fraction collector. However, no blood samples were obtained at 24 hours to assess longer-term changes in renal function.

At the end of the experiment, animals received intramuscular gentamicn (150 mg), and all catheters were removed.

### Effects of single (s-TP) or multiple (m-TP) doses of TP in septic shock

Baseline measurements were collected for a 120 minute control period before the induction of sepsis by intravenous infusion of live *Escherichia coli (E.coli)* (3×10^9^ colony forming units) over five minutes at midnight. Approximately 8 to 10 hours after the initial bolus, animals typically reached the pre-defined cardiovascular criteria for randomization (hyperdynamic sepsis): 10% decrease in MAP, 50% increase in heart rate and 30% increase in CO.

After reaching the criteria for septic shock, the sheep in this group (s-TP, n = 7) were observed for a 120 minute pre-intervention period before being randomly assigned to receive an intravenous bolus injection of either TP (1 mg) or vehicle (saline). MAP, CO, HR, vital organ blood flow (CBF, MBF, IBF and RBF) and urinary output were continuously measured for 6 hours after the injection of TP. After 2 weeks recovery, the sheep were crossed over to the other arm of the study.

A second group of sheep (m-TP, n = 6) followed the same protocol except that they received an intravenous bolus injection of TP (1 mg), followed by two bolus injections of TP (0.5 mg) at 2 hourly intervals.

Although, doses of 2 mg are often given to patients of 80–100 Kg in weight, concern that the standard human dose of 1 mg might be excessive in 35 kg sheep led us to also study 0.5 mg doses to improve clinical relevance. Additionally, to assess the impact of repeated dosing, which could not be addressed by single bolus administration, we studied the impact of two sequential boluses of terlipressin of 0.5 mg. We reasoned that in 35 kg sheep they might be the equivalent of two sequential boluses of 1 mg in man. The objective of these studies was to elucidate how these clinically common interventions may affect key regional circulations in mammals.

### Statistical analysis

The data in the table and text are presented as mean ± standard deviation for 2 hours. In the figures of hemodynamic changes, values are mean ± standard deviation over 20 minutes of the percentage change from the control values. Statistical analysis was performed using SAS version 9.2 (SAS Institute Inc., Cary, NC, USA). All outcomes were initially assessed for normality and found to have sufficient symmetry to validate the use of repeat measures mixed linear modeling with each sheep treated as a random effect. Main effects were fitted for time, intervention (vehicle, s-TP and m-TP) and an interaction between time and intervention to ascertain if interventions behaved differently over time. Whilst analysis was stratified by stage (control, sepsis and intervention), particular attention was focused on reporting main effects for the three interventions with post-hoc pairwise comparisons adjusted by a Bonferroni correction. A two-sided p-value of 0.05 was considered to be statistically significant.

## Results

**Figure 1:**
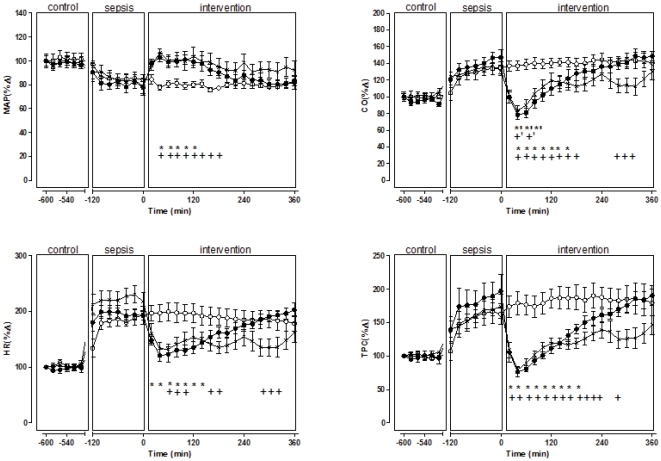
Systemic effects of a single bolus injection of TP multiple bolus injection of TP and vehicle in septic sheep. Single bolus injection of TP (black circles); multiple bolus injection of TP (crosses); and vehicle (white circles). Heart rate, HR; mean arterial pressure, MAP; cardiac output, CO; total peripheral conductance, TPC. Results are means ± SD. * p<0.05 for comparisons between s-TP and vehicle at each intervention point, + p<0.05 for comparisons between m-TP and vehicle at each intervention point, #p<0.05 for comparisons between m-TP and vehicle at each intervention point, ^*′^ p<0.05 for comparisons between intervention and control within s-TP, ^+′^ p<0.05 for comparisons between intervention and control within m-TP.

After the bolus injection of live *E.coli*, prior to the intervention with s-TP or vehicle, all sheep reached the predefined criteria for hyperdynamic sepsis within 10 hours. Hyperdynamic sepsis was indicated by a 23% fall in MAP (93±12 to 74±12 mmHg, p<0.05), peripheral vasodilatation (TPC increased from 45±7 to 80±28 mL/min/mmHg, p<0.05), a 40% increase in CO (4.0±0.3 to 5.6±0.8 L/min, p<0.05) a doubling in HR (73±11 to 144±9 beats/min, p<0.05), a 29% decrease in SV (55±9 to 39±5 mL/beats, p<0.05) a halving in urine output, a 60% decrease in creatinine clearance, a 37% increase in serum creatinine and a 300% increase in blood lactate levels (Table 1, Figure 1). All animals, however, survived the experiment.

**Table 1:** Renal functional and biochemical effects of terlipressin (TP) in severe sepsis.

Variable	Intervention	Control	Septic	TP 2 hours	TP 4 hours	TP 6 hours
**FF (%)**	s-TP	38.5±13.7	10.4±4.5	38.2±7.8	26.1±16.4	14.4±6.2
**FF (%)**	m-TP	32±9	6±5	22±7	15±8	15±2
**FF (%)**	vehicle	37.6±9.6	12.6±5.3	14.7±4.4	18.4±7.7	16.7±4.3
**UO (ml/h)**	s-TP	61±18	25±11	307±158	138±93	31±15
**UO (ml/h)**	m-TP	99±23	27±13	229±160	100±34	60±21
**UO (ml/h)**	vehicle	73±35	42±42	24±11	30±11	43±22
**p-Cr (µmol/L)**	s-TP	96±7	135±24	118±15	119±16	133±25
**p-Cr (µmol/L)**	m-TP	86±10	127±24	111±23	101±14	103±18
**p-Cr (µmol/L)**	vehicle	100±11	131±31	129±27	127±28	126±27
**CCr (ml/min)**	s-TP	62±18	24±8	85±20	62±39	34±15
**CCr (ml/min)**	m-TP	54±17	16±14	40±6	29±12	34±8
**CCr (ml/min)**	vehicle	64±18	26±14	31±13	37±12	37±11
**FENa (%)**	s-TP	0.38±0.21	0.35±0.23	2.52±1.61	1.46±0.96	0.31±0.23
**FENa (%)**	m-TP	0.45±0.38	1.33±1.27	2.26±1.42	1.45±0.64	0.66±0.50
**FENa (%)**	vehicle	0.57±0.31	0.52±0.41	0.19±0.12	0.22±0.19	0.19±0.19
**PaO2 (mmHg)**	s-TP	94.2±11.1	83.7±7.8	84.3±6.1	83.6±7.9	84.3±5.6
**PaO2 (mmHg)**	m-TP	100.1±9.3	90.4±3.3	94.4±6.5	94.7±8.2	92±5.1
**PaO2 (mmHg)**	vehicle	95.1±13.3	83.9±10.3	88.4±10.1	88.1±10.3	87.7±8.8
**PaCO2 (mmHg)**	s-TP	36.2±2.9	33.1±6.2	34.4±3.6	32.9±2.2	31.7±1.5
**PaCO2 (mmHg)**	m-TP	32.7±2.0	28±1.7	31.6±1.4	31.8±1.1	31.0±2.5
**PaCO2 (mmHg)**	vehicle	35.8±2.4	33.4±3.5	33.3±3.7	35.1±5.6	35.4±6.5
**HCO3 (mmol/L)**	s-TP	27.9±3.1	26.0±3.8	24.7±2.6	23.9±2.5	25.9±1.4
**HCO3 (mmol/L)**	m-TP	23.3±3.9	22.2±2.0	23.0±1.1	22.6±0.8	23.8±0.9
**HCO3 (mmol/L)**	vehicle	27.9±1.3	25.3±2.0	26.2±2.5	27.0±2.9	27.1±2.8
**Na (mmol/L)**	s-TP	144±2	145±2	147±3	146±4	144±4
**Na (mmol/L)**	m-TP	143±2	144±3	145±6	143±5	142±5
**Na (mmol/L)**	vehicle	144±4	144±3	143±3	143±4	143±4
**Cl (mmol/L)**	s-TP	111±4	110±4	109±2	111±4	109±4
**Cl (mmol/L)**	m-TP	111±3	113±3	110±6	111±5	111±4
**Cl (mmol/L)**	vehicle	111±5	114±5	109±3	109±4	106±4

Filtration fraction = FF; urinary output = UO; plasma creatinine = p-Cr; creatinine clearance = CCr; fractional excretion of sodium = FENa.

**Figure 2:**
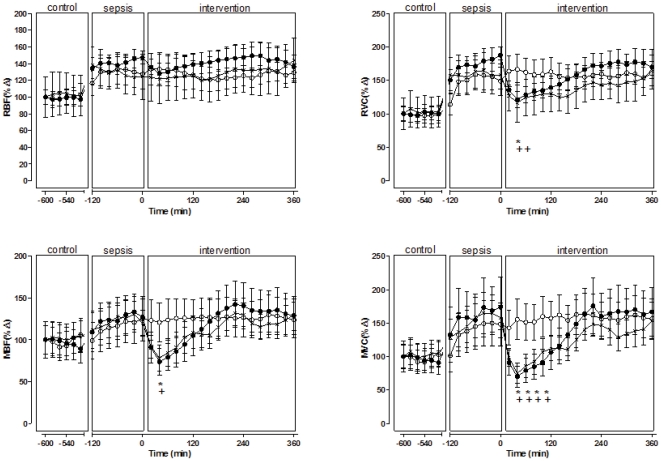
Effects on renal and mesenteric vasculatures of a single bolus injection of TP multiple bolus injection of TP and vehicle in septic sheep. Single bolus injection of TP (black circles); multiple bolus injection of TP (crosses); and vehicle (white circles). Renal blood flow, RBF; renal vascular conductance, RVC; mesenteric blood flow MBF; mesenteric vascular conductance, MVC. Results are means ± SD. * p<0.05 for comparisons between s-TP and vehicle at each intervention point, + p<0.05 for comparisons between m-TP and vehicle at each intervention point, *′ p<0.05 for comparisons between intervention and control within s-TP, ^+′^ p<0.05 for comparisons between intervention and control within m-TP.

**Figure 3:**
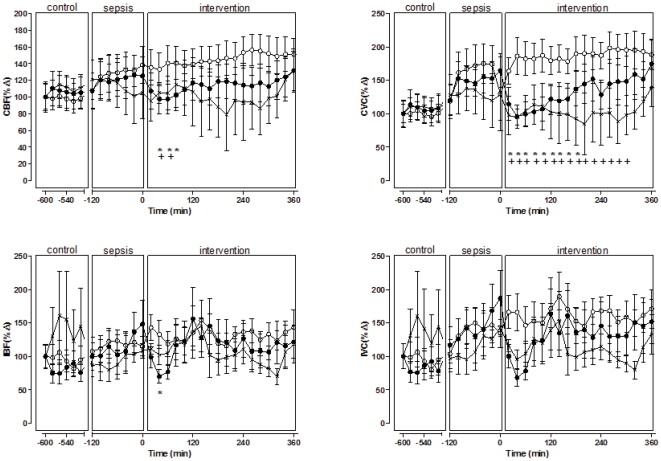
Regional effects on coronary and iliac vasculatures of a single bolus injection of TP multiple bolus injection of TP and vehicle in septic sheep. Single bolus injection of TP (black circles); multiple bolus injection of TP (crosses); and vehicle (white circles). Coronary blood flow, CBF; coronary vascular conductance, CVC; iliac blood flow IBF; iliac vascular conductance, IVC. Results are means ± SD. * p<0.05 for comparisons between s-TP and vehicle at each intervention point, + p<0.05 for comparisons between m-TP and vehicle at each intervention point, * p<0.05 for comparisons between intervention and control within s-TP, ^+,^ p<0.05 for comparisons between intervention and control within m-TP.

During severe sepsis there was vasodilatation in all vascular beds, as shown by the increases in each regional conductance. This led to significant increases in renal blood flow (RBF) and mesenteric blood flow (MBF) and similar trends for coronary blood flow (CBF) and iliac blood flow (IBF) (Figures 1, 2, 3).

### Responses to single dose TP (s-TP)

Single dose TP (s-TP) (1 mg) returned MAP to control levels (74±12 to 89±9 mmHg, p<0.0001), but the effect was temporary and dissipated within four hours. Single dose TP also caused transient decreases in HR, CO and TPC, but SV was not improved. In particular, CO significantly decreased to below control levels (Figure 1, Table 2). After s-TP, there was substantial but transient vasoconstriction in the mesenteric circulation and a significant decrease in MBF compared with vehicle (Figure 2, Table 2). In the coronary vasculature s-TP caused vasoconstriction and CBF significantly decreased compared with vehicle. IBF was unchanged (Figure 3, Table 2).

**Table 2:** Pair-wise comparisons for main effects during the intervention period ^.

Variable	Group1	Group2	% Difference	SE	P-value*
**CBF**	mTP	sTP	0.21	0.03	<0.0001
**CBF**	Vehicle	mTP	0.10	0.03	0.010
**CBF**	Vehicle	sTP	0.30	0.02	<0.0001
**CO**	mTP	sTP	−0.09	0.03	0.002
**CO**	Vehicle	mTP	0.27	0.03	<0.0001
**CO**	Vehicle	sTP	0.19	0.02	<0.0001
**CVP**	mTP	sTP	−0.22	0.05	<0.0001
**CVP**	Vehicle	mTP	0.74	0.05	<0.0001
**CVP**	Vehicle	sTP	0.51	0.04	<0.0001
**HR**	mTP	sTP	0.22	0.04	<0.0001
**HR**	Vehicle	mTP	0.08	0.04	0.11
**HR**	Vehicle	sTP	0.30	0.03	<0.0001
**IBF**	mTP	sTP	−0.18	0.07	0.04
**IBF**	Vehicle	mTP	0.45	0.07	<0.0001
**IBF**	Vehicle	sTP	0.27	0.05	<0.0001
**MAP**	mTP	sTP	0.08	0.01	<0.0001
**MAP**	Vehicle	mTP	−0.17	0.01	<0.0001
**MAP**	Vehicle	sTP	−0.09	0.01	<0.0001
**MBF**	mTP	sTP	0.12	0.04	0.002
**MBF**	Vehicle	mTP	−0.04	0.04	0.68
**MBF**	Vehicle	sTP	0.08	0.03	0.005
**RBF**	mTP	sTP	−0.16	0.04	<0.0001
**RBF**	Vehicle	mTP	−0.01	0.04	1.0
**RBF**	Vehicle	sTP	−0.17	0.03	<0.0001
**RVC**	mTP	sTP	−0.33	0.04	<0.0001
**RVC**	Vehicle	mTP	0.29	0.04	<0.0001
**RVC**	Vehicle	sTP	−0.04	0.03	0.56
**TPC**	mTP	sTP	−0.29	0.04	<0.0001
**TPC**	Vehicle	mTP	0.69	0.04	<0.0001
**TPC**	Vehicle	sTP	0.40	0.03	<0.0001

^ single dose terlipressin = sTP; multiple dose terlipressin = mTP; Group numbers indicate nature of comparison. A positive difference means that group one had a higher value than group 2. The number under %difference represents the percentage of difference between the two groups. A negative difference means that group 2 had a higher value. For example, mean arterial pressure (MAP) was higher with mTP or STP than vehicle. SE = standard error; CBF = coronary blood flow; CO = cardiac output; CVP = central venous pressure; HR = heart rate; IBF = iliac blood flow; MAP = mean arterial pressure; MBF = mesenteric blood flow; RBF = renal blood flow; RVC = renal vascular conductance; TPC = total peripheral conductance.

*All values represent P values after Bonferroni adjustment.

After s-TP, there was a transient and minor decrease in renal vascular conductance, but overall RBF increased due ot increased perfusion pressure (Table 2). There was also a large increase in UO over the first 2 hours following s-TP(Table 3).

**Table 3:** Pair-wise comparisons of terlipressin (TP) effects during the intervention period∧.

Variable	Group1	Group2	Difference	SE	P-value*
**CCr**	mTP	sTP	−24.38	5.37	0.0001
**CCr**	Vehicle	mTP	−0.82	5.37	1.0
**CCr**	Vehicle	sTP	−25.20	4.79	<0.0001
**Cl**	mTP	sTP	0.77	1.02	1. 0
**Cl**	Vehicle	mTP	−2.91	1.02	0.02
**Cl**	Vehicle	sTP	−2.14	0.78	0.03
**FENa%**	mTP	sTP	0.03	0.25	1.0
**FENa%**	Vehicle	mTP	−1.25	0.25	<0.0001
**FENa%**	Vehicle	sTP	−1.23	0.23	<0.0001
**FF%**	mTP	sTP	−9.35	2.37	0.0009
**FF%**	Vehicle	mTP	−0.33	2.37	1.0
**FF%**	Vehicle	sTP	−9.68	2.27	0.0003
**HCO3**	mTP	sTP	−1.46	0.67	0.10
**HCO3**	Vehicle	mTP	3.94	0.67	<0.0001
**HCO3**	Vehicle	sTP	2.49	0.56	0.0002
**K**	mTP	sTP	−0.41	0.13	0.008
**K**	Vehicle	mTP	1.06	0.13	<0.0001
**K**	Vehicle	sTP	0.65	0.10	<0.0001
**Lac**	mTP	sTP	−0.88	0.40	0.10
**Lac**	Vehicle	mTP	−0.55	0.40	0.51
**Lac**	Vehicle	sTP	−1.43	0.31	0.0001
**Na**	mTP	sTP	−2.85	1.05	0.03
**Na**	Vehicle	mTP	0.37	1.05	1.0
**Na**	Vehicle	sTP	−2.48	0.82	0.01
**PCO2**	mTP	sTP	−1.66	1.02	0.33
**PCO2**	Vehicle	mTP	3.76	1.02	0.002
**PCO2**	Vehicle	sTP	2.10	0.82	0.04
**P-Cr**	mTP	sTP	−18.20	6.53	0.02
**P-Cr**	Vehicle	mTP	22.20	6.53	0.004
**P-Cr**	Vehicle	sTP	4.00	5.28	1.0
**PO2**	mTP	sTP	8.50	2.22	0.001
**PO2**	Vehicle	mTP	−8.23	2.22	0.002
**PO2**	Vehicle	sTP	0.27	1.71	1.0
**UO**	mTP	sTP	−24.42	24.39	0.97
**UO**	Vehicle	mTP	−101.86	24.39	0.0004
UO	Vehicle	sTP	−126.29	21.68	<0.0001

∧ single dose terlipressin = sTP; multiple dose terlipressin = mTP; Group numbers indicate nature of comparison. A positive difference means that group one had a higher value than group 2. A negative difference means that group 2 had a higher value. SE = standard error; CCr  =  creatinine clearance in ml/min; Cl  =  chloride in mmol/L; FENa = fractional excretion of sodium as %; FF = filtration fraction as %; HCO3  =  bicarbonate in mmol/L; K = potassium in mmol/L; Lac = lactate in mmol/L; Na = sodium in mmol/L; PCO2 = partial arterial pressure of carbon dioxide in mmHg; P-Cr = plasma creatinine in µmol/L; PO2 =  partial arterial pressure of oxygen in mmHg; UO = urine output in ml/hr.

* All values represent P values after Bonferroni adjustment.

The sepsis-induced decrease in creatinine clearance (Ccr) was corrected by s-TP (Table 3). There were also increases in filtration fraction (FF) and fraction excretion of sodium (FENa) (Table 1).

**Figure 4:**
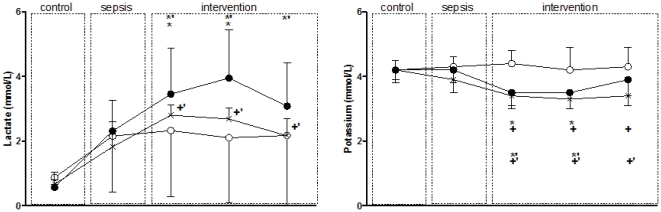
The effects on blood lactate and potassium of a single bolus injection of TP (1.0 mg, s-TP n = 7, black circles) multiple bolus injection of TP (1.0 mg+0.5 mg+0.5 mg, m-TP, n = 6, crosses) and vehicle (saline n = 7, white circles) in septic sheep. Data were collected during a control period, sepsis period and each 2 hour intervention period in conscious sheep with septic shock. Results are means ± SD. * p<0.05 for comparisons between s-TP and vehicle at each intervention point, + p<0.05 for comparisons between m-TP and vehicle at each intervention point, ^*^ p<0.05 for comparisons between intervention and control within s-TP, ^+,^p<0.05 for comparisons between intervention and control within m-TP.

Blood lactate was increased by the induction of sepsis and was increased further by treatment with s-TP (to 3.95±1.49 mmol/l) (Figure 4, Table 3). There was a significant reduction in serum potassium after s-TP (Figure 4).

### Responses to multiple doses of Terlipressin (m-TP)

Following administration of E.coli, all sheep reached the predefined criteria for sepsis, as described for the s-TP group (Table 1, Figure 1).

The first injection of TP (1 mg) returned MAP to the pre-sepsis control level, and the subsequent injections of TP (0.5 mg) at 2 hourly intervals increased MAP (Figure 1). The multiple injections of TP caused similar, but more sustained, reductions in CO and TPC than s-TP (Figure 1, Table 1).

Treatment with m-TP had differential effects on the regional vasculature. As with s-TP, the greatest degree of vasoconstriction occurred in the mesenteric and coronary circulations, with less effect in the renal vasculature and little effect on the iliac vascular bed. The effects on vasoconstriction in the mesenteric and coronary vasculature were more sustained than with s-TP, but they were still transient (Figure 2 and 3). Overall, during the period of observation, compared with sTP, mTP delivered increases in mean coronary and mesenteric blood flow, and MAP, while decreasing renal blood flow and total peripheral conductance (Table 1).

The initial injection of TP significantly increased UO, but despite further injections of TP there was a decrease in UO towards sepsis levels over the following four hours (Table 2 and 3). Administration of m-TP was associated with reduction in p-Cr, which was more sustained than with s-TP as was the increase in CCr (Table 1). Although FENa increased after the initial injection of TP, this effect was not maintained (Table 3). However FF was significantly greater with mTP than with sTP (Table 3) Finally, blood lactate increased and serum potassium decreased with both agents (Figure 4, Table 3). Despite, the increase in lactate, no episodes of coronary or gut ischemia occurred during or after the experiments.

## Discussion

### Key findings

In experimental severe hyperdynamic sepsis, a single bolus injection of TP restored MAP to pre-sepsis levels and improved renal function. However, TP also reduced CO by approximately 30% with slow return to control value over four hours, MBF by approximately 40% with slow return to control values by three hours and CBF by approximately 20%, an effect sustained over the six hour observation period, increased plasma lactate and lowered serum potassium. Treatment with multiple bolus injections of TP caused qualitatively similar effects but with significant attenuation of the s-TP induced beneficial renal effects and relative preservation of cardiac output.

### Comparison with previous studies

In patients with septic shock, AVP has been used as a vasopressor to maintain arterial pressure [2], [11], [12] in septic patients. Because of its longer half-life [11], [12], [14], TP has been given as a bolus injection (typically 1 mg) every 4–6 hours [14]. Although in patients with septic shock bolus injection of TP restores MAP, with improvement in CCr and UO [4], [9], [10], [15], it has a number of potential deleterious effects [6], [16], such as decreased oxygen delivery, and, in some cases, myocardial, splanchnic and digital ischemia [12], [17].

In our experimental animals, the systemic effects of TP were consistent with those seen in patients with septic shock [4], [9], [10] suggesting a degree of clinical relevance. Its temporary systemic effects are also consistent with previous experimental studies [18]. With repeated doses of TP, the effect on MAP appeared more sustained compared with s-TP indicating that it may be feasible to maintain a normal MAP in septic shock with frequent doses of TP. Although continuous infusion of low-dose TP in septic sheep and in patients with septic shock also maintained MAP [19]–[21], this approach would make TP almost identical to AVP in terms of hemodynamic and functional effects.

In clinical and experimental studies, single dose TP leads to decreased cardiac output and oxygen delivery [4], [10], [12], [15]. The similar change seen in our experiments might have contributed (together with mesenteric vasoconstriction) to the increase in blood lactate concentration. With multi-dose TP, there were no additional increases in lactate, suggesting that a multi-dose protocol or an infusion might decrease these unwanted effects. The large reduction in CO caused by TP is in accord with similar effects seen in this model of sepsis with AVP [22], and the well established ability of AVP to sensitize the arterial baroreflex, thus causing a greater fall in CO and HR for a given rise in pressure [23]. Importantly, all studies using single bolus TP in human and experimental sepsis have shown a decrease in CO [10], [15], [24], [25], [26], [27] of similar magnitude to what was found in our study. In the only study we identified that assessed the effect of multiple boluses [26] of 1 mg in sheep, the systemic hemodynamic findings, urine output findings and lactate effects were almost identical.

### Renal effects of Terlipressin

A single dose of TP temporary returned RVC towards control levels, without a change in RBF, and transiently improved renal function. With multiple doses of TP the hemodynamic effects and improvement in Ccr were attenuated but sustained, and the effect on urine output remained transient. Similarly, in patients with septic shock, bolus injection of TP or AVP improved Ccr [4], [10], [12], [15]. This effect may be explained by preferential vasoconstriction of the *efferent* arterioles, where there is a higher density of AVP V1 receptors [28]. This effect would lead to an increase in glomerular filtration pressure, Ccr and FF and decrease in RVC as seen in the present study. These effects of TP and AVP are similar to those of angiotensin II [29]. In addition, the increase in MAP caused by TP might have contributed to the increased UO and FENa. However, this effect was greater than that seen with equipressor doses of noradrenaline and adrenaline [30], [31], indicating that the improvement in renal function is not simply due to the increase in perfusion pressure. Finally, decreases in potassium observed after s-TP and m-TP are consistent with previous report of increased urinary excretion of potassium [32].

### The effects of Terlipressin on vital organ blood flow

TP has a potent splanchnic vasoconstrictor effect in non-septic states [33], [34], and a similar action has been proposed in sepsis [12], [35]. In these conscious animals with severe hyperdynamic sepsis, TP significantly decreased MBF suggesting that that, even in the hyperdynamic state, TP might induce excessive mesenteric vasoconstriction. In sepsis, a high coronary blood flow appears essential to preserve cardiac oxygen metabolism [36], [37]. Our findings that TP caused coronary vasoconstriction and rapidly reduced CBF in a model of sepsis suggests that in patients with hyperdynamic sepsis, and at risk of ischemic heart disease, treatment with TP should be used with caution to avoid excessive coronary vasoconstriction, myocardial ischemia and dysfunction [19], [38], [39]. There are several reports describing adverse vasoconstrictor effects of TP on lower limbs, digits and skin, resulting in amputations and skin necrosis [6], [11], [13]. In the present study, TP did not cause excessive vasoconstriction in the iliac vascular bed, but skin blood flow was not measured and no necrosis was observed during or following the 6 hour intervention period.

### Strengths and limitations

To our knowledge, this is the first study to report on the effects of a bolus of 1 mg of TP on mesenteric blood flow in awake hyperdynamic septic animals and to report on the effects of such bolus and repeated doses of TP on coronary, renal and iliac blood flow in septic animals. In addition, it is the first to provide controlled information on the effects of TP on renal function during hyperdynamic sepsis. Finally, our model of hyperdynamic sepsis simulates the typical clinical human phenotype suggesting a degree of clinical relevance. However, this study has several important limitations. We did not measure the effect of TP on oxygen extraction because of the difficulty of chronically maintaining patency of venous sampling cannulae. We did not measure the effect of TP on all aspects of vital organ function. However, the information of stroke volume provides a clinically relevant measure of its cardiac effect, lactate blood levels provide a clinically relevant indirect assessment of splanchnic function and urine output, serum creatinine levels, filtration fraction and fractional excretion of sodium all provide a clinically relevant assessment of kidney function. We did not study the effects of continuous TP infusion, we considered we would assess the effects of TP when given in its most commonly prescribed and manufacturer recommended dosage schedule first. Although delayed response to TP has been reported, the majority of studies of TP report a rapid response (24–48 hours). Thus, short-term changes in hemodynamics are likely representative of the early effect of TP in general. We can however, make no comment on the longer term effects of the drug.

### Conclusions

In experimental hyperdynamic severe sepsis, treatment with TP returned MAP to control levels, increased renal vascular resistance and improved renal function. However, it also decreased CO by approximately one third, mesenteric and coronary blood flow by similar percentages, and caused hyperlactatemia and hypokalemia. These data imply that caution should be applied when prescribing TP in septic patients at risk of coronary or mesenteric ischemia; increase our understanding of the regional hemodynamic effects of TP in severe sepsis and assist clinicians in their selection of optimal vasopressor support for patients with severe sepsis and septic shock.
